# Effects of clomiphene citrate plus estradiol or progesterone on endometrial ultrastructure: An RCT

**DOI:** 10.18502/ijrm.v18i3.6718

**Published:** 2020-03-29

**Authors:** Robabeh Taheripanah, Maryam Kabir-Salmani, Masoomeh Favayedi, Marzieh Zamaniyan, Narges Malih, Anahita Taheripanah

**Affiliations:** ^1^Infertility and Reproductive Health Research Center, Shahid Beheshti University of Medical Sciences, Tehran, Iran.; ^2^Department of Biomaterials and Tissue Engineering, Stem Cell Division, National Institute of Genetic Engineering and Biotechnology, Tehran, Iran.; ^3^Department of Obstetrics and Gynecology, Infertility Center, Mazandaran University of Medical Sciences, Sari, Iran.; ^4^Diabetes Research Center, Mazandaran University of Medical Sciences, Sari, Iran.; ^5^Social Determinants of Health Research Center, Shahid Beheshti University of Medical Sciences, Tehran, Iran.; ^6^Department of Molecular and Cellular Sciences, Faculty of Advanced Sciences and Technology Pharmaceutical Sciences Branch, Islamic Azad University, Tehran, Iran.

**Keywords:** Ovulation induction, Clomiphene, Estradiol, Progesterone, Electron microscopy, Endometrium.

## Abstract

**Background:**

Pinopods concentrations in endometrial surface is a marker of implantation. Estradiol valerate (EV) was used to change the adverse effects of Clomiphene Citrate (CC) on the endometrium.

**Objective:**

The goal was to assess whether there is a significant difference in the endometrial pinopods concentrations and other parameters after adding EV and progesterone to higher doses of CC.

**Materials and Methods:**

In this prospective randomized clinical trial, a total of 30 women who did not respond to 100 mg of CC from February 2016 to June 2016 were evaluated. They were divided into three groups: group I) received 150 mg of CC alone, group II) CC with EV, and group III) CC plus progesterone. On day 21 of the menstrual cycle, endometrial biopsy, a blood sampling, and a scanning by electron microscopy were performed.

**Results:**

On day 21 of the menstrual cycle, there was no significant difference in the pinopods concentrations (p = 0.641) and serum estrogen levels (p = 0.276) between groups. However, the Serum progesterone levels in group I was higher than the other two groups (p = 0.007) in the same day.

**Conclusion:**

Since the addition of EV and progesterone to higher dosages of CC did not change the pinopods concentration and serum estrogen levels on day 21 of the menstrual cycle, and the serum progesterone levels was higher in CC alone group (i.e. group I) compared to other groups, it can be concluded that the anti-estrogenic effects of CC just appear on the endometrium and not on the plasma levels.

## 1. Introduction

Induction of ovulation is one of the first steps of infertility treatment among infertile couples who have polycystic ovarian syndrome, or other causes of infertility such as unexplained infertility. Clomiphene citrate (CC) is a known and proven first-line treatment for ovulation induction (1). Data confirms that ovulation rate with this method is about 80%, but pregnancy rates are about 40% (2).

Different factors such as unexplained infertility might cause the lower rate of pregnancy after successful induction, but it should be noted that anti-estrogenic effects of CC on the endometrium can lead to disturbances in endometrium during implantation. This effect is dose-dependent and further increases in dosage makes this problem more visible (3). Estrogen can cause endometrial cell proliferation and also increase the cells surface microvillus, change the cell volume, and increase progesterone receptors; this can all support the hypothesis regarding the effect of exogenous administered estrogen during ovulatory cycle on pregnancy rate (4). Progesterone can cause the uterine glands to become wider and more complex and it also might increase their activity (5). To reduce this effect, researchers have used other drugs such as tamoxifen as ovulation-stimulating agent believing it might have less anti-estrogen effect on the endometrium and less impact on the cervical score (6).

In some studies, addition of conjugated estrogens in the second half of the follicular phase to compensate for the anti-estrogenic effect of clomiphene has been evaluated (7), but no study was done at the cellular levels and endometrial morphology. Pinopods are bubbles-like projections found on the apical surface of the endometrial epithelium. These organelles are some micrometers wide and protrude into the uterine cavity over the microvilli level. Electron microscopy is the main instrument used to illustrate these organelles. Pinopod expression is restricted to a short-term period of maximal two days in the menstrual cycle presumably in the implantation window. The pinopod expression pattern all over the menstrual cycle supports their use as markers of implantation. HOXA-10 is part of a homeobox gene that its expression is essential for endometrial receptiveness to blastocyst attachment. However, it has a crucial role in pinopod growth (8). Undeniably, suppressing HOXA-10 leads to reduction in the number of pinopods. In some study, CC leads to a decrease in HOXA-10 and may cause lower pregnancy rate (9).

Therefore, in this research, we tried to examine pinopod concentration in the CC-treated group alone compared with adding estradiol or progesterone to see whether these changes in pinopod concentrations could be reversed by hormonal intervention. Our hypothesis was that adding estradiol valerate (EV) and progesterone to higher doses of CC could improve endometrial morphology such as pinopods concentration assessed by electron microscopy and other factors affecting implantation, so that the addition may improve implantation compared to CC alone.

## 2. Materials and Methods

### Patients

**Figure 1:**
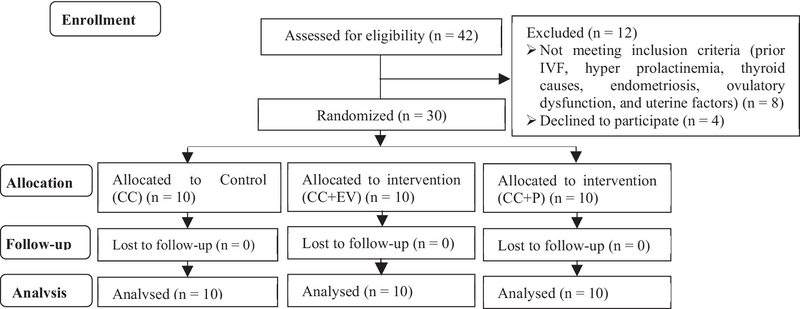
Flowchart of eligible patients.

Thirty infertile patients were randomly divided into three groups from February to June 2016 (n = 10/each). The inclusion criteria were woman aged ≤ 35 yr and regular menses (25-34 days), and unexplained infertility for at least 1 yr. All patients had normal serum levels of thyroid-stimulating hormone, and prolactin. Also, all participants had a history of unsuccessful usage of CC 100 mg/day in prior cycles. The exclusion criteria were previous in-vitro fertilization or intra-cytoplasmic sperm injection, any other causes of infertility such as hyper prolactinemia, thyroid causes, endometriosis, ovulatory dysfunction, and uterine factors. The flowchart of eligible patients has been shown in Figure 1.

### Ultrastructure of pinopods 

In the present study, three methods of ovulation induction in infertile patients were used. For the effect of drugs on pinopods concentration and embryo implantation the endometrium was evaluated at the mid-luteal phase (time of implantation). The first group received 150 mg of CC (Aboraihan Company, Tehran, Iran) alone on days 5-9 of menstrual cycle. The second group in addition to 150 mg of CC on days 5-9 of menstrual cycle received EV (Aboraihan Company, Tehran, Iran) for five days from day 8 of menstrual cycle at a dose of 4 mg daily. In the third group, in addition to clomiphene on days 5-9 of menstrual cycle, medroxyprogesterone acetate (Aboraihan company, Tehran, Iran) 10 mg daily was prescribed from day 14 until the time of biopsy on day 21 of the menstrual cycle (II).

After the documentation of dominant follicles by the ultrasound in ovaries, 10,000 IU/l HCG (Pregnyl, Aboureihan, Tehran, Iran) was prescribed to release the oocysts (10, 11). Then, on day 21 of the menstrual cycle (seven days after the administration of HCG), endometrial biopsy was performed using Novak curette to take the samples from the anterior fundal section of the uterus. We also took blood samples for the measurement of serum progesterone. Quantitative evaluation of uterine dome density using scanning electron microscopy was performed as previously described (12). In summary, biopsy samples were washed immediately in phosphate buffered (0.1mol/l, PH 7.4) (PBS tablets, USA, MP Company) and were then transferred to 2.5% Glutaraldehyde (Germany, DSM company) for initial fixation. Then, the samples were transferred to a solution of 1% osmium tetroxide (Germany, Ridel de Haen) as secondary fixation for at least 1 hr. Thereafter, the samples were dehydrated in a graded series of ethanol (5, 50, 70, 90, 99%) (Razi company, Iran), dried by machine (Polaron CPD 7501 system (VG Microtech, UK), and then mounted and covered by gold in a Bio-Rad SC510 sputter coater (VG Microtech).

Finally, the number of uterine domes in experimental and control groups were counted, at the same magnification in six random fields. Thus, 30 random fields in each group were assessed morphologically and statistically trying to obtain samples from similar regions of uterus (anterior-fundal wall of the uterus) and in a specific time of cycle (on day 21). Pinopods were evaluated as sphere-shaped lumps without microvilli on the apical surface of the endometrium and were semi-quantitatively evaluated as lacking (0), isolated pinopods (+), small clusters of pinopods (++), and feeder pinopods (+++). To perform this electron microscopy by ZEISS EVO Scanning Electron Microscope (SEM) (Germany, Carl Zeiss Company, EVO 40), was used, and the number of pinopods that were larger than 0.2 mm were counted per mm.

### Ethical consideration

This study was a prospective randomized clinical trial, which was conducted at Imam Hossein Hospital, a tertiary referral university center. The research protocol was approved by the Institutional Review Board and ethics committee of the Shahid Beheshti University of Medical Sciences, Tehran, Iran (SBMU.RAM.REC.1386.16). Informed consent was obtained from all patients before starting the procedure.

### Statistical analysis

In this pilot study, 30 patients were randomly divided into three groups. We enrolled 10 patients in each group, which gave the study a statistical power of 80% at alpha level 0.05. Descriptive statistics are used to describe the basic features of the data in a study. The data analysis was done using SPSS for windows, version 21.0 (IBM Corp., Armonk, NY). The Chi-square test was used to compare frequencies. Because of the small sample size, quantitative data in three groups were analyzed using the Kruskal-Wallis analysis. P < 0.05 was considered statistically significant.

## 3. Results

There was no significant difference between groups regarding the mean age (26.11 ± 3.37, 26.81 ± 2.85, 27.00 ± 4.30 respectively, p = 0.766). Also, there was no statistically significant difference regarding pinopods concentrations, serum estrogen levels on day 21 of the cycle and the endometrial thickness, number and size of ovarian follicles on day 14 of the cycle among the three groups. The serum progesterone level on day 21 of menstrual cycle among patients treated with CC was higher than the other two groups (34.98 ± 20.35 vs.8.87 ± 7.02 vs. 12.00 ± 9.52, respectively, p = 0.007) (Table I).

**Table 1:** Comparison of cycle's characteristics and pinopods ultrastructure and hormonal profile between the three groups of patients


**Variables**	**Group I${}^{a}$**	**Group II${}^{b}$**	**Group III${}^{c}$**	**P-value**
Age (yr)*	26.11 ± 3.37	26.81 ± 2.85	27.00 ± 4.30	0.766
Pinopod concentration on day 21 (Pinopod ultrastructure)*	27.71 ± 21.85	39.42 ± 28.99	27.69 ± 19.05	0. 723
Serum estradiol levels on day 21*	243.24 ± 69.53	194.55 ± 152.58	262.12 ± 77.00	0.276
Endometrial thickness on day 14*	7.55 ± 1.74	7.87 ± 1.39	8.00 ± 1.85	0.903${}^{\#}$
Serum progesterone levels on day 21*	34.98 ± 20.35	8.87 ± 7.02	12.00 ± 9.52	0.007${}^{\#}$
Number of follicles ≥ 17 mm on day 14**	8 (88.9%)	8 (72.7%)	7 (87.5%)	0.577${}^{\$}$
*Data presented as Mean ± SD, ** data presented as n(%); ${}^{\#}$ ANOVA; ${}^{\$}$ Chi-square test was used ${}^{a}$ Clomiphene citrate (CC) alone, ${}^{b}$CC plus estradiol valerate (EV), ${}^{c}$CC plus progesterone				

## 4. Discussion 

In this study, there was no significant difference in the number of ovarian follicles greater than 17 mm in ultrasound on day 14 of the menstrual cycle among patients treated with CC alone (group I) compared to those treated with CC plus EV (group II) and CC plus progesterone (group III). This finding is supported by other studies that have observed comparable efficacy when using CC or CC plus estradiol regarding the number and size of ovarian follicles in patients undergoing intrauterine insemination (13, 14). However, Gerli and Unfer and co-workers examined different forms of estrogen as ethinyl estradiol and phytoestrogens (13, 14), which are different from us. Also, they did not investigate progesterone supplementation effects. In our study, there was no difference among patients in group I compared to those in groups II and III regarding the endometrial thickness on day 14 of the menstrual cycle. Similar results have been reported by Check *et al* (15). It may be due to higher estrogen secretion by multiple follicular maturations on day 14 that could compensate anti-estrogenic effects of CC. Other researchers have found a decrease in endometrial thickness in clomiphene alone when they compared group I with group II (15-17). It could be due to the suppression of endometrial receptivity markers (HOXA10 and integrin alpha (v) beta (3)) in rat by CC and the significant differences between ovulation and pregnancy percentage (18). Nevertheless, these authors investigated more number of patients that may explain the different results. Also Unfer *et al*, reported an increase in endometrial thickness following the addition of 1,500 mg daily phytoestrogens in 65 women with oligo menorrhea or amenorrhea who were undergoing CC plus IUI (14). Yet, they used different form and different dose of estrogen in other type of population, which could explain the different results. In recent study, there was no significant difference in serum estradiol levels on day 21 of menstrual cycles between the three groups, but statistically significant differences were observed in progesterone levels on day 21 in group I versus groups II and III. Contrary to our study is one previous research about CC treatment versus no treatment in infertile women by Palomino *et al*. They demonstrated that estradiol and progesterone levels in mid-luteal phase was higher in CC treated groups than the spontaneous endometrium but there was no differences in the of plasma estradiol and progesterone levels between out-of-phase and in-phase endometrium (19). These results verify an assumption that plasma levels of steroids are independent from endometrial growth in (20). We did not find a significant difference considering the pinopod concentrations on day 21 between the three groups. Although, Pinopod concentration was slightly higher in group II, it wasn't significant. Ganesh and co-workers found that when patients with unexplained infertility were treated with letrozole or CC, endometrial receptivity markers such as αvβ3 integrin, L-selectin, leukemia inhibitory factor (LIF), and pinopod concentrations were meaningfully higher compared to controls (21). However, they examined unexplained infertility and didn't use another hormonal supplementation such as estradiol or progesterone that may induce different effects. Cortínez *et al*. showed complete expression of pinopode throughout the implantation window of letrozole-treated group. They found a small development in endometrial maturation in letrozole group, which could be described by the estrogen/progesterone ratio, which favors progesterone in therapeutic cycles (22). Pinopods is progesterone dependent. The relationship between mid-luteal progesterone rise and the first presence of pinopods during the menstrual cycle was well-established (23).

This is also in line with the study by Omran *et al*. that evaluated the effect of CC on uterine artery blood flow using pulsed Doppler and endometrial vascularization using 3D power Doppler in unexplained infertility and found lower endometrial flow in the stimulated cycles, but there was no difference in serum estradiol and progesterone concentrations between natural and stimulated cycles (24) similar to us. However, we did not use ultrasonography to access the endometrium. Therefore, it can be investigated in future researches among the three mentioned treatment groups.

In one previous study, CC had shown a direct influence reducing the pinopods formation in the mid-luteal phase (16, 12). In a recent study, pinopods concentration slightly increased with adding EV to CC. It seems that induced pinopods concentration by adding estradiol to CC or use of letrozole may lead to better implantation and pregnancy rates (1, 23, 25). In one study, researchers have examined the distribution of the L-selectin ligand (MECA-79) in human endometrial apical membrane area during the implantation window. We found higher expression of MECA-79 in pinopods in mid-luteal phase compared to the utero dome-free areas. These finding suggests a new role for endometrial pinopods similar to endothelial docking structures in tethering process, which permits the leukocytes to move on the endothelial cell wall (26). Since no other study has been performed to compare pinopods concentrations, further studies with larger sample size are needed to better evaluate these variables and recognize the effects of hormonal supplementation on endometrial receptivity markers as pinopods. In this study, there were higher levels of serum progesterone on day 21 of the menstrual cycles in the CC alone compared to the other two groups. It is, therefore, essential to perform more extensive studies with larger sample sizes.

## 5. Conclusion

There was no superiority of adding estradiol or progesterone supplementation to clomiphene citrate for enhancing pinopod concentration and endometrial thickness or serum estradiol levels in women who didn't response to clomiphene (Clomiphene failure). Yet, pinopod concentrations is slightly increased with adding estradiol which could be examined more again in future researches. Thus, it can be concluded that the anti-estrogenic effects of CC just appear on the endometrium not on the plasma levels.

##  Conflict of Interest 

The authors declare that there are no potential conflicts of interest.
